# Protocol for a randomised controlled trial to estimate the effects and costs of a patient centred educational intervention in glaucoma management

**DOI:** 10.1186/1471-2415-12-57

**Published:** 2012-11-22

**Authors:** Heidi Cate, Debi Bhattacharya, Allan Clark, Richard Fordham, Caitlin Notley, David C Broadway

**Affiliations:** 1 Ophthalmology, Norfolk & Norwich University Hospital NHS Foundation Trust, Colney Lane, Norwich, NR4 7UY, UK; 2 School of Pharmacy, University of East Anglia, Norwich, NR4 7TJ, UK; 3 Norwich Medical School, University of East Anglia, Norwich, NR4 7TJ, UK

**Keywords:** Adherence, Compliance, Glaucoma, Ocular hypotension medication, Motivational interviewing, Behaviour change counselling, Travalert® dosing aid

## Abstract

**Background:**

Poor glaucoma education is thought to be a causative factor of non-adherence to glaucoma therapy. However, the multi-factorial nature of non-adherent behaviour may explain the failure of purely educational interventions to achieve significant improvement in adherence. Behaviour Change Counselling (BCC) allows both the imparting of information and assessment of patient ambivalence to medication use and may elicit behaviour change in order to achieve better adherence. The chronic and complex nature of glaucoma means that patient non-adherence to glaucoma therapy does not easily correlate with measureable objective clinical endpoints. However, electronic medication monitoring offers an objective method of measuring adherence without reliance on clinical endpoints.

**Methods/design:**

The study is a randomised controlled trial (RCT) with glaucoma (open angle) or ocular hypertension patients attending a glaucoma clinic and prescribed travoprost. The study will determine whether additional glaucoma education using BCC is beneficial and cost effective in improving adherence with glaucoma therapy. An 8-month follow-up period, using an electronic adherence monitoring device (Travalert® dosing aid, TDA), will indicate if the intervention is likely to be sustained in the longer term. Additionally, a cost-effectiveness framework will be used to estimate the cost benefit of improving adherence. The development of a novel intervention to deliver glaucoma education using BCC required practitioner training and fidelity testing. Five practitioners were successfully trained to become Glaucoma Support Assistants able to deliver the BCC intervention. The research group had prior clinical and investigative experience in this setting, and used multiple strategies to design a method to address the study objectives.

**Discussion:**

This RCT, using BCC to improve adherence to ocular hypotensive therapy, to our knowledge is the first within this disease area. Using a variety of adherence measures allows examination of the known inaccuracies of patient self-report with respect to glaucoma medication. The novel BCC component has undergone fidelity testing using BECCI and the BCC template will ensure conformity to a standardised intervention.

**Trial registration:**

Current Controlled Trials: ISRCTN89683704

## Background

Untreated glaucoma is a chronic, progressive condition leading to visual field loss and risk of blindness. The majority of glaucoma damage is preventable with appropriate therapy [1-3] and the mainstay of treatment is eye drops to reduce intraocular pressure (IOP) [4]. Non-adherence to therapeutic regimens is associated with a reduction in treatment benefit [5] resulting in additional health service costs through changes to prescribed medication requiring additional follow-up of efficacy, wastage of unfinished pharmaceutical supplies, or the costs of surgery that may have been avoidable [6]. If surgical treatment is required, there is also the increased risk of associated adverse effects.

Patient non-adherence with anti-glaucoma therapy is poorly understood with reported rates between 5% and 80% [7]. Historically, non-adherence has been categorised as intentional or unintentional, but the ultimate behaviour is often an amalgam of these factors [8]. Unintentional non-adherence is a passive process that prevents use of medication as intended by the prescriber, such as forgetfulness, poor comprehension of dosing regimen or physical inability to self-administer medication. Intentional non-adherence is a deliberate decision by the patient to deviate from the prescribed recommendations by not taking medication, reducing the dosing frequency or premature discontinuation.

According to UK national guidance [4], a typical care pathway involves referral to a specialist glaucoma clinic for diagnosis by standard glaucoma examination, followed by long-term monitoring, with treatment if indicated, according to risk of disease progression. Provision of information for patients, carers and family members is usually offered by the diagnosing clinician.

Poor glaucoma education has been cited as an explanation for non-adherence to therapy [9-17] however, the magnitude and nature of any association is not consistent. A semi-structured educational session reported by Sheppard et al. [9] identified an improvement in participant knowledge but no significant difference in adherence between intervention and control groups. Sheppard’s findings may, in part, be attributable to the small sample size (n = 73), short follow-up (12 weeks) and failure to ascertain the fidelity of intervention delivery by nurses.

Studies related to oral anti-hypertensives have found that education alone is ineffective in improving adherence [18,19] and similar outcomes have been reported with glaucoma. Three studies using education alongside an individualised care planning programme have demonstrated an improvement in adherence [11,16,17].

A study of a 30 minute education and tailoring program (n=73) reported by Norell et al. [11], detected a positive, significant improvement in adherence. Norell’s educational component was similar to other reported studies, however, the additional ‘one to one’ tailoring program allowed the patient to consider and discuss strategies for incorporating the medication administration into daily activities; the study findings were limited by a short follow-up (20 days), no estimate of sustainability of intervention effect and no estimate of intervention cost.

One method to overcome the issue of assuring fidelity is to use standardised information. A web-based glaucoma education and support intervention study [20], used links to information on the internet for participants to access independently. Whilst information provision was standardised, participants controlled how much and what information they chose to access based upon personal requirements. The study failed to significantly improve adherence [20] which may partly be attributable to the absence of direct contact with healthcare team members, as non-adherent participants are more likely to feel that insufficient time is devoted to them by their healthcare team [21].

It appears that the influence of both intentional and unintentional factors and complexities of non-adherent behaviour may explain the failure of purely educational interventions to achieve significant adherence improvement. A Cochrane review of adherence interventions [22] concluded that effective adherence interventions were complex in nature which may explain the success of the Norell et al. education and tailoring program [11,22] relative to simple educational interventions. The Norell et al. approach of using glaucoma assistants rather than written or IT based techniques is also supported by research which has demonstrated that interventions using personal contact are more effective [23]. It seems appropriate, therefore, to conduct a larger scale trial adopting a similar approach to Norell et al. with longer follow-up, and detailed reporting of research methodology to demonstrate research rigour.

A further appropriate enhancement for future interventions would involve considering all factors known to affect adherence. The Theory of Planned Behaviour suggests that the best predictor of behaviour is intention which is determined by the attitude towards the behaviour formed from beliefs about the likely outcomes of behaviour and the value of that outcome [24]. Similarly, Social Cognitive Theory considers the key constructs of a particular behaviour to be patient belief in their capability to perform the required behaviour (self-efficacy) and their perception that the behaviour will have a positive effect on the health condition (outcome expectation) [25]. When patients express ambivalence to adhering to their prescribed regimen, Motivational Interviewing (MI) and more recently Behaviour Change Counselling (BCC) have been developed in order to identify ambivalence and guide patients in adopting behavioural change [26].

MI is defined as ‘a client-centred, directive method for enhancing intrinsic motivation to change by exploring and resolving ambivalence’ and has been successfully applied to improving medication adherence [27-30]. MI in its original format consisted of multiple sessions, each of 30–60 minutes. However, in a medical setting, patient encounters usually range from 10–15 minutes and patients often do not see the same clinician throughout follow-up, limiting the use of MI. Additionally, in medical and public health settings, there is often a multi-component approach to care such as the provision of educational materials and information communication [27]. Thus, Behaviour Change Counselling (BCC), an adaption of MI suitable for brief consultations, was developed for use in healthcare. Whereas MI uses open questions and reflective listening [31], BCC can additionally be used to exchange information. BCC can be of brief duration or extended to a longer time if required, typically 5–30 minutes [32].

There are a multitude of adaptations to the original form of MI used in randomised controlled trials (RCTs). However, RCTs rarely report precise intervention details and there is often no evidence of any skill assessment of the practitioners delivering the intervention or measures of patient-centeredness [33]. A number of instruments measure patient-centeredness [34] but these are not specific to health behaviour change techniques. The Motivational Interviewing Skill Code (MISC) [35] and Behavioural Change Counselling Index (BECCI) [33] are specific measures of fidelity to MI and BCC respectively. MISC applies three phases of analysis by means of direct observation of a recorded clinical encounter. MISC scores from each phase are computed to produce an overall summary score which can be compared to the benchmarks depicting proficiency in MI techniques [35]. Despite revision in 2008 to a more streamlined process [36], MISC still includes a number of subsections that are not essential for users of BCC [33].

BECCI was developed to evaluate practitioner competence in using BCC in controlled trials. BECCI focuses on the practitioner consulting behaviour, rather than patient response, in a single-phase analysis using Likert-type scales to indicate either frequency or strength of practitioner behaviour over 11 items to produce an overall mean score [33].

There are numerous approaches to measuring adherence, each with merits. Patient self-report, using questionnaires, diaries and/or interviews are cheap and easy to administer but in comparison with objective measures, can yield higher adherence estimates [37]. This discrepancy is attributed to both the social desirability to be adherent resulting in reporting bias and memory bias. Prescription refill databases can provide adherence estimates based on how many prescriptions have been ordered over a period of time, which may show intention to use medication, but does not ensure administration [38].

In diseases where medication adherence correlates with therapeutic outcome, objective observations are possible. However, with glaucoma, adherence does not easily correlate with immediate clinical benefit and ideally both adherence and clinical endpoints should be assessed [39]. The chronic nature of glaucoma means that determination of the relationship between adherence magnitude and therapeutic efficacy requires long term follow-up with assessment of optic nerve damage and/or visual field loss. IOP may provide an alternative measure, however, there is considerable diurnal variation [40-42] and so there is no universal standard for using IOP as an adherence measure [43].

An 80% adherence rate is widely recognised as ‘acceptable’ for many systemic medications [44] but there is no such acceptance for ocular hypotensives. However, a recent cross-sectional glaucoma study in the USA found that participants with adherence rates < 80%, according to a medication event monitoring system (MEMS®), had worse visual field defects than those with adherence rates ≥ 80% [45].

Electronic drug monitoring is a more objective method to measure adherence and particularly appropriate with eye drops as ‘pill counting’ methods cannot be employed. Electronic monitors are, however, often expensive and cumbersome, limiting their use. The Alcon® Travatan dosing aid (TDA; Travalert®, Alcon, Fort Worth, TX) is designed for patients to easily reload their eye drop bottle into the device and electronically records every drop discharged [46]. Whilst electronic monitoring devices have previously been criticised for modifying adherence and/or behaviour by emphasising the monitoring itself (the Hawthorne effect) [47], a pilot study has established that the TDA had no such demonstrable impact [48].

## Methods/design

The purpose of this study was to determine whether additional education and advice about glaucoma using a BCC intervention, improves adherence with topical anti-glaucomatous therapy. The objectives were to:

● Determine the effect of the intervention on adherence.

● Determine the longevity of any intervention effect on adherence.

● Estimate the incremental cost-effectiveness ratio (ICER) of improving adherence.

● Describe user experiences of the educational intervention and self perception of adherence in order to gain a better understanding of the components of the intervention that might improve adherence.

● Report any predictors of non-adherence based upon social, demographic, medical and family history information.

### Intervention development

**Figure 1:**
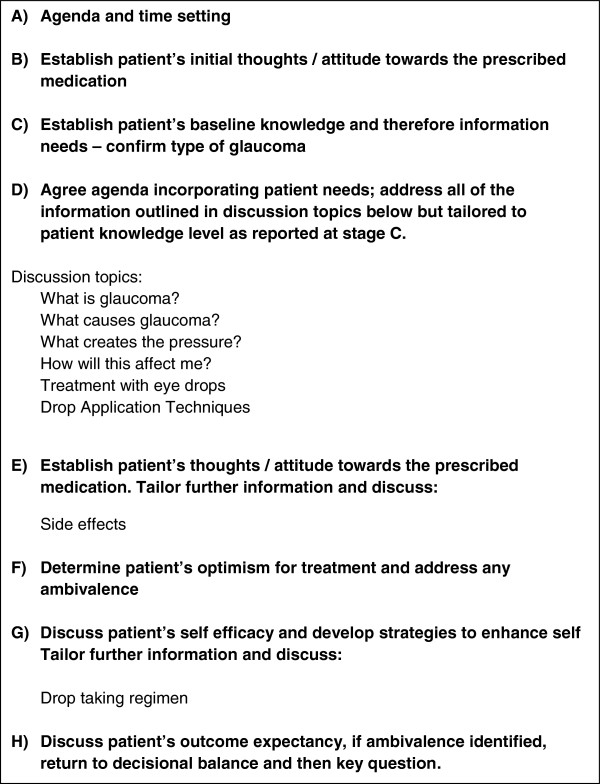
Behavioural change counselling template.

**Figure 2:**
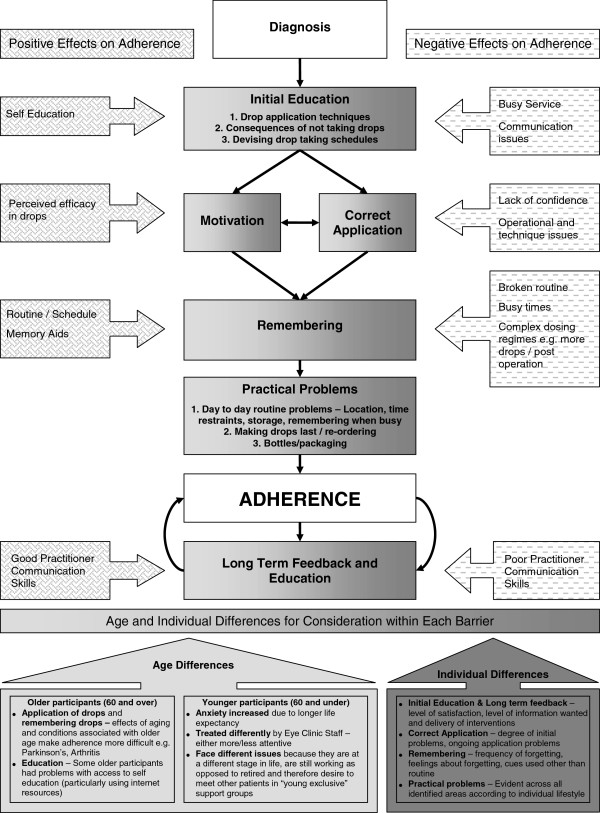
The glaucoma medication adherence model.

The intervention was standardised by the development of a BCC template (Figure 1) which was informed by the following:

● the Glaucoma Medication Adherence Model which was derived from a qualitative study exploring patient perceived barriers to adherence with glaucoma medication (Figure 2) [13];

● glaucoma and ocular hypertension education requirements identified from the literature and expert clinician advice;

● the Satisfaction with Information about Medicines Scale (SIMS) which has been demonstrated to be a reliable predictor of adherence [49]. SIMS was used to ensure that the BCC template contained the standard information deemed necessary for new glaucoma patients to make an informed decision about use of their ocular hypertensive treatment (travoprost).

#### Glaucoma support assistant training

Five Glaucoma Support Assistant (GSAs) roles were created for the purpose of this study. Prior to recruitment, the GSAs had worked in varying roles within the eye department (glaucoma research technician, ophthalmic nurse, glaucoma nurse specialist (2) and glaucoma research co-ordinator), all with experience of working with glaucoma patients. Five GSAs were utilised to ensure the results of the study were attributable to the intervention method rather than the specific context of any one individual delivering the intervention. The GSAs participated in BCC training and quality assurance sessions. Training session one (4 hours duration), included a two hour introduction to the causes of non-adherence and predictors of behaviour. For the remainder of the session, the GSAs were introduced to MI theory and participated in role-play sessions facilitated by a qualified MI coach who provided appropriate feedback. A two month interval was then allowed to enable the GSAs to practice the MI and BCC skills within their usual clinical duties. At training session two (three hours duration), the GSAs reported any problems encountered with training session one after implementation. Individualised guidance was provided in response to any reported problems and the remaining time devoted to role-playing observed by the MI coach, with feedback provided.

The GSAs also underwent a training session with the Glaucoma Specialist Consultant to ensure competency in the knowledge and skills required to deliver education to patients about glaucoma and related issues. The GSAs had the freedom to conduct the counselling session in their own BCC style. Education was tailored to individual patients, but followed the BCC template (Figure 1).

#### Fidelity testing

Six months after training completion, GSA information provision was assessed in terms of adherence to the BCC template and consultation style assessed using BECCI [33] via a video recorded session with an actor-patient (see Table 1 for criteria). A ‘brief’ was prepared for the role-playing actor by the Glaucoma Specialist Consultant and MI coach. The overview of the character to be played by the actor-patient provided the GSAs with an opportunity to identify ambivalence to adherence arising from the non-curative nature of the treatment and lifestyle barriers.

**Table 1:** BECCI scoring of role play assessment

**BECCI criterion**	**GSA 1**	**GSA 2**	**GSA 3**	**GSA 4**	**GSA 5**
**Domain 1. Agenda setting and permission seeking**					
1. The practitioner invites the patient to talk about behaviour change	1.5	2.0	2.0	2.1	1.7
2. The practitioner demonstrates sensitivity to talking about other issues	2.3	2.0	2.7	3.0	2.0
**Domain 2. The why and how of change in behaviour**					
3. Practitioner encourages patient to talk about current behaviour or status quo	2.3	2.3	2.7	2.7	3.0
4. Practitioner encourages patient to talk about behaviour change	2.0	2.0	2.3	2.7	2.7
5. Practitioner asks questions to elicit how patient thinks and feels about the topic	2.0	2.3	3.0	3.0	3.0
6. Practitioner uses empathic listening statements when patient talks about the topic	2.0	3.0	2.7	3.0	2.7
7. Practitioner uses summaries to bring together what the patient says about the topic	2.3	1.0	0.7	2.3	2.0
**Domain 3. The whole consultation**					
8. Practitioner acknowledges challenges about behaviour change that the patient faces	1.3	2.3	2.7	2.0	1.7
9. When practitioner provides information, it is sensitive to patient concerns and understanding	2.7	2.7	3.0	3.0	2.3
10. Practitioner actively conveys respect for patient choice about behaviour change	1.3	2.3	2.3	1.7	1.7
**Domain 4. Talk about targets**					
11. Practitioner and patient exchange ideas about how the patient could change current behaviour	2.3	2.0	2.0	2.3	2.0
**Practitioner BECCI score**					
Mean score excluding domain 1	2.1	2.2	2.4	2.6	2.3

The video recorded role-play sessions were independently reviewed according to the BECCI criteria by the MI coach and two experts in MI independent to the research study. Table 1 summarises the BECCI scores by the three assessors. The mean average BECCI score reveals that the GSAs used behaviour change counselling (BCC) to some extent during the simulated session; GSA consultation compliance was 100% for 11 of the 16 BCC criteria. Three GSAs did not clearly discuss with the patient the fact that there was no tangible effect from using the eye drops. Two GSAs did not discuss the patient’s expectations of adherence to treatment and for each of the following criteria, one GSA did not clearly address the issue during the role-play: eye drop application technique, patient’s thoughts/attitudes regarding the prescribed medication, patient’s optimism and perceived self-efficacy regarding adherence to the prescribed travoprost. Individualised, written feedback was provided to each of the GSAs.

#### GSA experiences of training and videoed actor-patient session

Glaucoma Support Assistants were invited to participate in a focus group. The aim of the focus group was to gain an understanding of GSAs experiences of the preparation and training prior to undertaking the study. The focus group lasting one hour was audio recorded and conducted by a moderator and assistant independent to the research study. The recording was transcribed verbatim and the data analysed using ‘Framework’ analysis [50,51].

Three of the five GSAs participated. Of the two GSAs that did not participate, one was the study facilitator and part of the management team and thus not invited in order to reduce bias, and the other member consented but was absent due to illness.

Of all the training given to the GSAs, the ‘acting session’ was discussed the most during the interview; this is understandable given the GSAs described it as “a terrifying experience”, and felt “really tense” prior to filming. For this reason, the fidelity test was far from a ‘normal’ case scenario and may not be a true representation of the interaction between participants and the GSA in the study setting. Using an actress in a simulated setting prohibited the fidelity test measuring true patient-practitioner behaviour in a naturalistic setting; this could be the reason why good information provision was evident during the role-play, but less emphasis placed on establishing ambivalence to eye drop use and the possible effects, as the GSAs did not feel they were addressing a ‘real patient’.

### Study design

**Figure 3:**
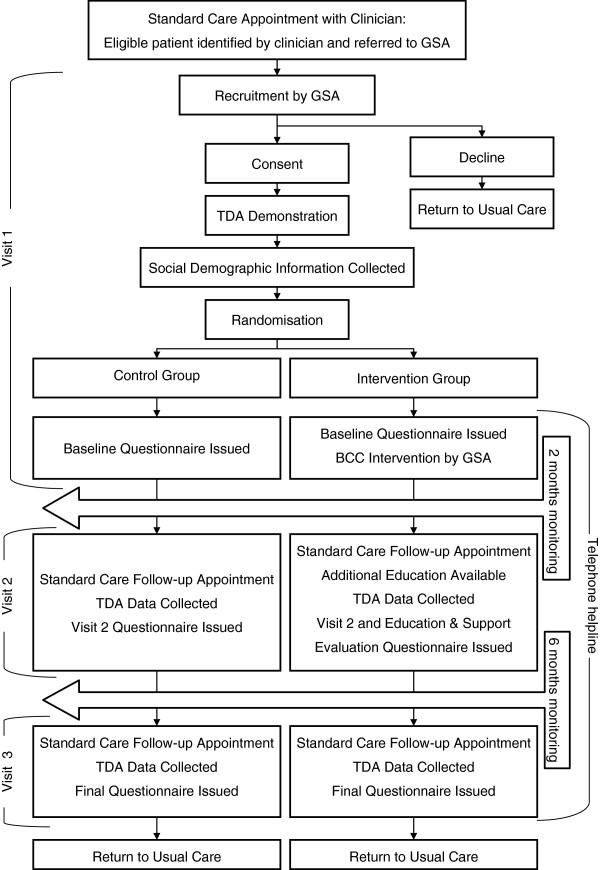
Illustration of the participant study pathway.

The study was a randomised controlled trial. A flow-chart of the study design is shown in Figure 3. The study received ethical approval from the Norfolk Research Ethics Committee, UK.

#### Setting, recruitment and treatment allocation

The study was conducted in the Glaucoma Clinic at the Norfolk and Norwich University Hospital, UK. Patients newly diagnosed, or previously untreated, with either glaucoma or ocular hypertension requiring the standard first line treatment of monotherapy with travoprost, (using established standard criteria as documented in the European Glaucoma Society Guidelines) [52], were invited to participate. Patients were >18 years of age, able to give informed consent, and had adequate ability to read and understand English. Patients requiring care-home staff or home-help (not provided by a co-habiting partner or family member) to apply eye drops were excluded.

Eligible patients were randomised using an automated telephone randomisation system to ensure allocation concealment. Randomisation was stratified by diagnosis (either glaucoma, or ocular hypertension/glaucoma suspect) and experience of the glaucoma service (new patient or follow-up patient but first intention to treat). Stratification controlled for possible variances, since previous studies have shown that patients with suspected glaucoma or ocular hypertension without the presence of manifest glaucomatous disease are less adherent than those with evidence of manifest glaucoma [53,54]. Also, patients previously reviewed as out-patients but not started on ocular hypotensive treatment, had an increased opportunity to ask questions or self-educate before therapy initiation and becoming eligible to participate in the study. Patients declining study participation, following consent, had demographic information collected (age, gender and index of multiple deprivation [IMD]).

Once randomised, participants were followed through to completion of the study regardless of whether travoprost treatment was continued or other additions/changes to the therapy were made.

#### Control group

Standard care was provided which involved attendance to a specialist glaucoma clinic for treatment initiation. Initiation included undergoing appropriate tests and a consultation of approximately 10 minutes with a specialist glaucoma clinician. The consultation consisted of the following:

● A brief explanation about glaucoma or ocular hypertension

● A summary of the proposed future management

● Guidance regarding drop administration

● The relevance of glaucoma with respect to driving and future vision.

A patient information leaflet providing information about glaucoma in general was provided.

#### The intervention group

In addition to standard care, the intervention group received the BCC intervention following the 10 minute consultation. A telephone advice-line for patients and their carers to respond to glaucoma related queries was also provided by GSAs.

#### Outcome and data collection strategies

A summary of the data collection process is provided in Table 2.

**Table 2:** Data collection process

**Method**	**Data collected**
**Baseline patient visit to Glaucoma Clinic**	
Structured interview	Medical history and social demographics (including: age, gender, IMD, education, employment, marital status, family history of glaucoma)
Baseline Questionnaire (Completed at home)	Satisfaction with Information about Medicines
Resources log	Time spent with GSA (in minutes)
Medical notes	Intraocular pressure measurement
**Visit 2**	
Visit 2 Questionnaire (Completed at home)	Satisfaction with Information about Medicines
	Morisky Medication Adherence Score
	Frequency of Missed Dose Score
	Possible predictors of adherence. e.g. do you apply your own drops or does somebody help you?
GSA Evaluation Questionnaire (for intervention group only and completed at home)	Satisfaction with Education and Support from GSAs
Resources log	Time spent with GSA (in minutes)
	Time spent with Clinician (in minutes)
Medical notes	Intraocular pressure measurement
**Visit 3**	
Visit 3 Questionnaire	Satisfaction with Information about Medicines
	Morisky Medication Adherence Score
	Frequency of Missed Dose Score
	Possible predictors of adherence. e.g. do you apply your own drops or does somebody help you?
Resources log	Time spent with GSA (in minutes)
	Time spent with Clinician (in minutes)
Medical notes	Intraocular pressure measurement
**Duration of study**	
Resources log	Time spent with GSA on help-line telephone calls
Evaluation of TDA data	Percentage adherence score using TDA
Prescription data capture	Repeat prescription count

#### Primary outcome measure

**Figure 4:**
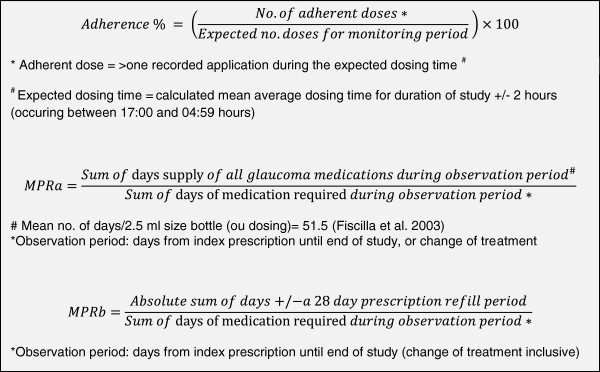
Calculations used for outcome measures; Adherence % and Medication Possession Ratios (a and b).

Adherence magnitude: Participant adherence to travoprost, was measured using the TDA. Participants were provided with written operating instructions [48] and shown how to use the TDA, including how to replace an empty bottle with subsequent supplies. Participants were asked to use the TDA to administer their travoprost eye drops for the study duration. The primary outcome measure was the average number of doses administered over the monitoring period, recorded by the TDA, and percentage adherence calculated using the adjusted adherence calculator [48] (calculation as in Figure 4). Adherence was calculated for the total study duration and for 8 separate monthly periods to explore trends throughout follow-up. Additionally, a binary classification of adherence score was used (‘adherent’ if the average number of TDA recorded doses is ≥ 80% of expected and ‘non-adherent’ if < 80%).

#### Secondary outcomes

Patterns of adherence behaviour: The TDA data were also used to produce graphical representations of adherence in order to classify patterns of adherence behaviour. The methodology and categories described by Ajit et al. in a 3 month study [55] and data on file, were used to classify 5 patterns of adherence; (i) discontinuation of dosing after a short time interval, (ii) adherence ≥ 97%, (iii) adherence < 97% ≥ 80%, (iv) frequent drug holidays, and (v) variable with frequent missed doses.

Medication Possession Ratio (MPR): Upon study completion, computer-generated repeat prescription information was collected from participants’ General Practitioner surgeries. Two MPR calculations were performed to indicate over- or under-usage of eye drop refills during the study; MPRa [38] used the average travoprost drop count [56] and MPRb, used the UK general prescribing instruction to renew eye drop prescriptions every 28 days (Figure 4).

Self-reported adherence: Two self-report adherence measures were utilised, (i) the Morisky Measure of Adherence Score (MMAS) [57] which is a four-item validated measure and (ii) the Frequency of Missed Dose (FMD) which is a measure developed by the authors to quantify the number of eye drop doses missed (once a day, once a week, once a month, or never).

Satisfaction with information received: The validated SIMS was used to quantify satisfaction with information received about travoprost [49].

Patient IOP: Routinely measured and recorded IOP results using Goldmann applanation tonometry were obtained for each eye, at each visit, from the participants’ medical records. The IOP measurement for each treated eye was used to calculate the % reduction in IOP from baseline, visit 2 and 3.

#### Predictors of adherence

Information about baseline socio-demographic characteristics and the following possible predictors of adherence were collected using a structured interview; a positive family (parent, sibling or offspring) history of glaucoma, previous use of eye drops, use of other prescribed medication and if this was used at the same time as travoprost, and self-administration of drops or help given by a family member. Co-morbidity was classified using the Charlson Co-morbidity Index [58] from information available in participant medical records.

#### Health economic measures

The health economic analysis was based upon the NHS perspective of using public funds in order to deliver free healthcare at the point of access. The primary outcome measure was incremental hospital cost per percentage gain in adherence. The specific costs associated with secondary care ophthalmic activity were identified on review of the patient pathway by an expert clinician (DCB), glaucoma health care professional (HC), and health economic advisor (RF). These costs were captured in resource logs and included; The time GSAs spent with each participant to deliver the BCC intervention and support the telephone helpline; on-costs were included in the hourly calculation of cost for each GSA according to the UK pay scale for NHS employees [59]. The cost of training GSAs in BCC were added assuming such training is renewed triennially due to staff turnover or updating skills and knowledge.

1. Clinician costs; calculated using the Unit Costs of Health and Social Care methodology [60]. The cost elements included were basic salary, salary on-costs and on-going training. An hourly clinician-cost, weighted by percentage of time spent in clinical practice was calculated and applied to the time spent with clinician as recorded in the resources log. Local service overhead costs were also factored in. Time spent with clinician rather than the local unit service cost was chosen to reflect the potential difference in consultation time between the two treatment arms. All follow-up appointments with clinicians or specialist nurses were recorded in the resource log and costed using the relevant standard local unit cost.

2. Consultation room servicing costs.

3. Additional glaucoma related standard care procedures; included in the cost analysis if in the opinion of two independent reviewers (HC and DCB), they were directly related to glaucoma management.

4. Prescription refill costs; British National Formulary costings for 2010 [61] were used.

Costs related solely to the study were not included, but a log maintained of the items necessary to conduct the study (available from corresponding author). The resource log was utilised to calculate the intervention cost for each participant.

A cost effectiveness analysis was undertaken to report the additional cost of delivering a BCC intervention in relation to the potential benefits of increased medication adherence. The analysis included costs from the point of randomisation to completion for each participant (pre-randomisation ophthalmic care and initial consultation costs were not included into the cost analysis).

### Rationale for sample size calculations

The absence of an accepted adherence rate with once daily glaucoma medication [7] and limited research to indicate the likely effect of an intervention led to estimates being derived from general medicine. In general medicine, average non-adherence rates of 25% have been reported [62] with interventions achieving an average 35% adherence increase [23]. Reports of glaucoma adherence studies using medication monitors, have had shorter follow-up time and more complicated dosing regimens than the proposed study [63,64]. Therefore, without a comparable study, a 20% increase in adherence was estimated for sample size power calculations with adherence defined as ≥ 80% of expected doses recorded by TDA. Assuming an adherence rate of 60% in the control group and 80% in the intervention group, 81 people in each group provides 80% power to detect a difference using a Chi-Squared test, at a 5% level of significance. Based on an estimated 20% drop-out rate, a target was set of 200 participants.

### Proposed analyses

Descriptive statistics will be used to characterise the demographics of the study population. All analyses will be based on the intention-to-treat principle. Missing data will be imputed using a multivariate normal imputation model after suitable transformations to ensure that the variables are normally distributed. A total of 10 imputed datasets will be created, each analysed separately and the results averaged using Rubin’s equations. The primary analysis will compare the percentage of “adherers” at 8 months post randomisation between intervention and control using chi-squared. Additionally, the combined month 7 and 8 post randomisation adherence score will be analysed using a *t*-test to compare the intervention and control group. A repeated measures analysis-of-variance will be carried out (with time measured in months) to assess for any difference between intervention and control over time.

Secondary analyses will include a sensitivity analysis of the primary analysis by adjusting for any baseline differences and for possible predictive variables (e.g. severity of disease) using logistic regression. SIMS, MPR and IOP change between 8 months and initial visit will be analyzed using a *t*-test to compare the intervention and control group. A reduction in IOP will be correlated with the percentage adherence rate. A correlation between self-reported adherence and the TDA adherence score will be carried out in each arm separately using a Spearman’s correlation coefficient. The self-reported adherence and SIMS scores will be compared both within the control and intervention groups to assess any changes in perceived quality of information given between the baseline, 2 month and end of study visits, as well as between the two groups to compare any differences. No subgroup analyses are planned.

Logistic regression will be used to identify possible adherence predictors. If appropriate, a multivariate model will be constructed to identify independent predictors of adherence.

The difference in total costs and adherence percentage will be calculated incrementally between the intervention and control group to provide the ICER of additional adherence from the intervention. A deterministic sensitivity analysis will be performed on the base case ICER using patient variability in cost and outcome to determine the robustness of the economic analysis. If there is a high degree of uncertainty, the analysis will be bootstrapped.

## Discussion

This article provides the description and rationale of an RCT to investigate the use of an educational intervention including BCC. The intention is to improve adherence to ocular hypotensive therapy and to our knowledge is the first in the UK within this disease area. The novel BCC component has undergone fidelity testing using BECCI and the BCC template to ensure conformity to a standardised intervention.

Adherence studies can be fundamentally biased by the selection of patients who attend follow-up appointments since non-adherent patients are more likely to drop out of follow-up care [22]. Thus, caution must be taken when extrapolating the results of adherence studies. The present study has made provision for this by recruiting at treatment initiation and monitoring over the duration of two follow up visits. The stratified randomisation also controlled for possible variances in the type of patients recruited.

The use of multiple adherence measures allows examination of both intentional and unintentional non-adherence, as prescription refill, FMD and TDA data provide a quantitative estimate of non-adherence whilst the MMAS provides some information about the reason for the non-adherence. Correlating self-reported adherence with the TDA helps to investigate the known inaccuracies of patient self-report with respect to glaucoma medication. Using prescription refill data to calculate the MPR should reveal participants who use more than one drop when instilling medication, since they will require more frequent refills and will have an MPR>1. The additional MPR calculation based upon period of time elapsed between refills will detect participants who do not order enough medication to supply them for 28 days as directed on the medication instruction label and will have an MPR <1.0.

The TDA, provides an objective measure of adherence which has previously been piloted by the research team. Whilst three previous studies have reported that the TDA accurately records drop administration [37,46,55], the Norwich pilot study highlighted potential problems in TDA operation by patients [48]. Following the pilot study, patient approved operating instructions were devised to avoid operational bias and ensure optimal use of the device. Additionally, an adjusted adherence calculator has been piloted to overcome the known limitations of the Travalert® Software which calculates the percentage adherence rate [48]. This adjusted adherence calculator enables a graphical representation of adherence behaviour patterns. The benefit of differentiating between different patterns of non-adherence which are not evident using global indices of adherence have previously been demonstrated by Ajit et al. [55]. This study will use graphical representation of adherence over an 8 month period, for the first time, to identify where further education or support may help improve long-term adherence to glaucoma medication.

There is little evidence to suggest that adherence interventions can consistently improve adherence to medication within the resources available in clinical settings. Interventions are generally led by research teams which cannot easily be translated into clinical practice [39]. However, this study will provide the economic costs associated with the provision of an adherence intervention led by specialist nurses and allied health professionals working within the local hospital and costs will be based upon local sources to provide a more realistic consideration of associated costs.

## Abbreviations

BCC: Behaviour change counselling; BECCI: Behaviour change counselling index; FMD: Frequency of missed dose; GSA: Glaucoma support assistant; ICER: Incremental cost-effectiveness ratio; IMD: Index of multiple deprivation; IOP: Intraocular pressure; MI: Motivational interviewing; MISC: Motivational interviewing skill code; MMAS: Morisky medication adherence scale; MPR: Medication possesion ratio; RCT: Randomised controlled trial; TDA: Travalert dosing aid; SIMS: Satisfaction with information about medication scale.

## Competing interests

The authors’ declare that they have no competing interests.

## Authors' contributions

HC, DB, DCB, AC, RF, RH conceived of the study, and participated in its design and coordination and helped to draft the manuscript. AC also performed the statistical analysis. RF also advised on the health economic analysis. CN participated in the design and analysis of the qualitative aspects of the study and helped to draft the manuscript. All authors read and approved the final manuscript.

## Pre-publication history

The pre-publication history for this paper can be accessed here:

http://www.biomedcentral.com/1471-2415/12/57/prepub
